# Same Clinical Reality of Spontaneous Rupture of the Common Iliac Artery with Pseudoaneurysm Formation—Comparison of Two Therapeutical Solutions, Endovascular Stent-Graft and Open Surgical Correction, for Two Cases and Review of the Literature

**DOI:** 10.3390/jcm12020713

**Published:** 2023-01-16

**Authors:** Horațiu Moldovan, Robert Tiganasu, Lucian Câlmâc, Cristian Voica, Marian Broască, Camelia Diaconu, Vlad Ichim, Mihai Cacoveanu, Liliana Mirea, Claudia Nica, Costin Minoiu, Irina Dobra, Daniela Gheorghiță, Lucian Dorobanțu, Adrian Molnar, Luminița Iliuță

**Affiliations:** 1 Faculty of Medicine, Carol Davila University of Medicine and Pharmacy, 014461 Bucharest, Romania; 2 Department of Cardiovascular Surgery, Emergency Clinical Hospital Bucharest, 014461 Bucharest, Romania; 3 Academy of Romanian Scientists, 54 Spl. Independentei, 050711 Bucharest, Romania; 4 Faculty of Materials Science and Engineering, Politehnica University of Bucharest, 060042 Bucharest, Romania; 5 Faculty of Medicine, Titu Maiorescu University, 040441 Bucharest, Romania; 6 Department of Cardiovascular Surgery, Monza Metropolitan Hospital, 040204 Bucharest, Romania; 7 Faculty of Medicine, Iuliu Hateganu University of Medicine and Pharmacy, 400012 Cluj Napoca, Romania; 8 Department of Cardiovascular Surgery, Heart Institute, 400001 Cluj Napoca, Romania

**Keywords:** iliac artery aneurysm, pseudoaneurysm, stent-graft

## Abstract

The incidence of isolated iliac artery aneurysms is approximately 2% and common iliac artery pseudoaneurysms are even rarer. A pseudoaneurysm is a localized hemorrhage as opposed to an actual aneurysm, which affects the entire vascular wall. They are typically asymptomatic and only detectable accidentally while looking for other causes. If large and symptomatic, they typically exhibit pressure symptoms as a result of the compression of the structures around them. Common symptoms include generalized stomach pain, urological problems, gastrointestinal bleeding, and neurological symptoms such as leg paralysis or sciatica-like back pain. Rarely, they may exhibit hemodynamic instability together with an aneurysm rupture, which has a high fatality rate. Due to the unique presentation, the diagnosis is typically rarely made and there is little experience with treating it. We report two cases of common iliac artery pseudoaneurysm found in two patients who had no notable medical history and who we chose to repair through the endovascular technique in the first case, an approach that has gained more ground for vascular repair worldwide, making it the current go-to method, and for the second case we chose a more traditional approach, through open surgery.

## 1. Introduction

Cases of isolated true iliac artery aneurysms are rarely reported in the literature. Even less common are those describing iliac artery pseudoaneurysms. The mechanism may be correlated with arteriosclerosis, trauma, surgical or interventional procedures, infection, connective tissue disorders, vasculitis, inflammation, and erosion secondary to malignancy [1,2,3,4,5,6]. The spontaneous formation of pseudoaneurysms originating from iliac arteries is extremely rare. Rupture is common in patients, with iliac artery pseudoaneurysms being associated with a high mortality rate [7,8,9,10]. Open surgery is the traditional method for treating pseudoaneurysms and is still frequently used. In the presence of local mass effects and in cases where interventional treatment has failed, open surgery is considered the method of choice. Many recommend it in patients with infected pseudoaneurysms, but the infection is not an absolute contraindication to endovascular techniques and management should be tailored to every case [11,12,13,14,15]. Surgical treatment disadvantages, such as long hospitalization time, requirement of general anesthesia, impaired wound healing, and an association with a higher morbidity and mortality rate, have shifted interest towards endovascular procedures. Over the past few years and with technological developments, interventional radiological treatment has gained popularity in the treatment of pseudoaneurysms [16,17,18,19,20,21,22,23,24]. Endovascular repair of iliac artery pseudoaneurysms is today a safe procedure in experienced centers, resulting in a decreased length of hospitalization, lower requirement for perioperative blood products administration, and similar intermediate-term outcomes as open repair, but the method may be difficult to implement in emergency settings [25,26,27,28]. Although there is no general consensus when discussing the optimal repair of pseudoaneurysms, a very useful option would be the combined approach, when stenting is possible, but in our case, we opted for the open approach when there was inadequate landing zone for the stent, and in the case of the endovascular repair, the dimensions of the hematoma had no significant impact on the adjacent structures, so there was no need to drain it. Another viable solution would be kissing stents for experienced centers, but our center is just starting to implement the endovascular repair; therefore, we believe that such an approach should be taken into account in adequate cases.

## 2. Case Reports

### 2.1. Case Report I

A hypertensive 77-year-old male, with no significant past medical history, presented to the Emergency Department with a 2-day history of left flank pain and no stool passage. Physical examination revealed apyrexia, no abnormalities of the cardiovascular system, a tender abdomen spontaneously which augmented on palpation in the left flank and the hypogastric region, and normal bilateral peripheral pulses. He was thought to have a subocclusive syndrome and was admitted to the general surgery unit for further investigations.

Blood tests revealed a normal white cell count, a hemoglobin level of 14 g/dl, a Hit level of 42.7%, with a slightly increased platelet level of 558,000, normal liver and renal parameters, and normal coagulation parameters. The bacteriological screening revealed a pharyngeal Klebsiella pneumoniae. An ECG showed a normal sinus rhythm with 60 bpm, with no significant modifications. A chest X-ray showed some diffusely bilateral interstitial micronodular opacities, without any other particularities. The plain abdominal radiography showed a left flank hydroaeric level as well as some air in the transverse colon and at the level of splenic flexure.

The abdominal and pelvis ultrasound revealed a left flank mass extending paraumbilical, with a hyperechoic area of 98/58 mm and a hypoechoic lateral extension of 75/67 mm. Echocardiography showed normal cavities, a good systolic function of the left ventricle (LVEF = 50%) and right ventricle, no significant valvular modifications, and a slightly dilated ascending aorta (38 mm).

**Figure 1:**
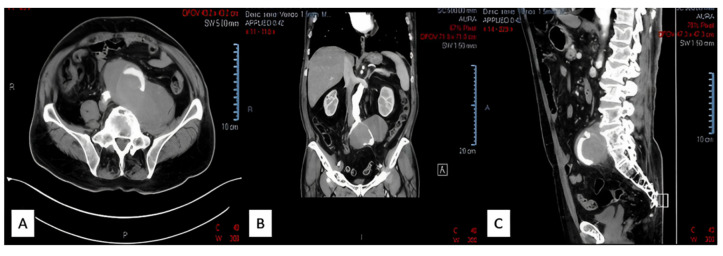
Left common iliac artery saccular aneurysm view: (**A**)—transversal; (**B**)—coronal; (**C**)—sagittal.

**Figure 2:**
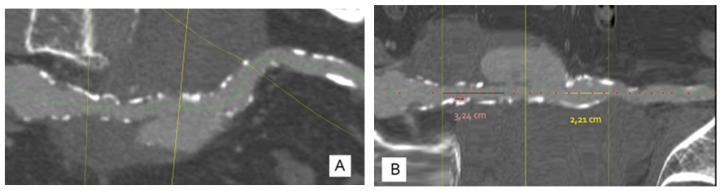
(**A**)—Curved MPR reconstruction showing the vascular axis with the aneurysm surrounding the AIC, with a limited entrance gate (yellow line in the middle); (**B**)—straightened reconstruction in the axis of the vessel (AIC); lines A and C represent the position of the bifurcation of the aorta and the bifurcation of the AIC, respectively, revealing the proximal landing zone at 32 mm and the distal landing zone at 22 mm.

A contrast-enhanced CT scan of the abdomen and pelvis presented in Figure 1 revealed a left common iliac artery saccular aneurysm (5.7/5 cm axial diameters and 6.5 cm cranio-caudal extension) with partial thrombosis, with circulating peripheral lumen of 1.1 cm, a periaortic hematoma (15/7.8 cm axial and 9 cm cranio-caudal) that pushed the left psoas muscle, extending towards the limbo–sacral vertebrae and partially compressing the right common iliac artery. It also showed a second smaller right common iliac artery (1.5/1.3 cm axial and 1.8 cm cranio-caudal extension) with partial thrombosis. Bilateral distal arterial axes of the lower limbs were permeable. No signs of rupture were present. There was no visualization of the left common iliac vein. The multiplanar reconstruction (MPR) is presented in Figure 2.

The patient was transferred in our clinic and was proposed for endovascular treatment. CTA was used to determine the needed stent and we consulted with a proctor who supervised the procedure. The measurements revealed that an Ovation Alto 80/160 mm stent was needed with an adequate landing zone.

**Figure 3:**
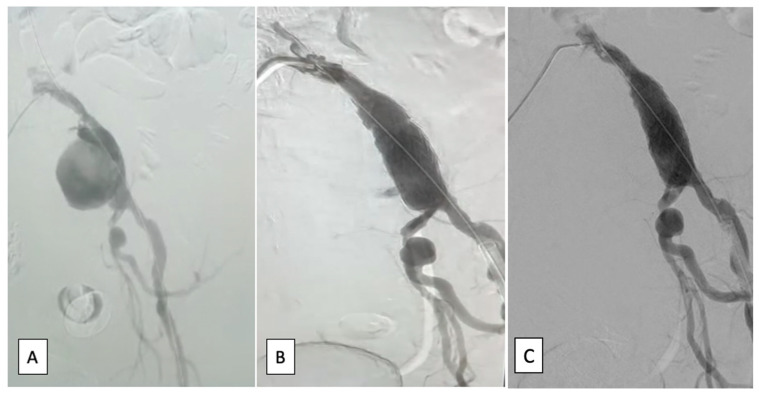
Angiographic image: (**A**)—periprocedural, left common iliac artery aneurysm; (**B**)—intermediate image with excluded left common iliac artery aneurysm and the implanted stent graft and leak; (**C**)—excellent end result.

The procedure was performed under local anesthesia. The left common femoral artery was isolated and through a 6F sheet, a stiff guide wire was advanced into the descending aorta; the right common femoral artery was isolated and through it was advanced a diagnostic catheter into the origin of left common iliac artery. A Proglide suture was placed at the level of the left common femoral artery puncture. The stent-graft (Ovation Alto 80/160 mm) was introduced and positioned under fluoroscopic guidance into the left common iliac artery. Distal endoleak was observed which needed balloon dilation (Coda balloon, 4 inflations with 12–16 mL). The final result was favorable. Local hemostasis was performed by tying the Proglide suture (on the left side) and a 6F Angioseal (on the right side), with good results. Periprocedural and postprocedural angiographic images are presented in Figure 3.

The patient’s evolution was favorable, with no need of ICU monitoring, normal bilateral femoral pulses, and no puncture bleeding. He was discharged in good condition on postprocedural day 2.

### 2.2. Case Report II

A 63-year-old male with numerous cardiovascular risk factors (hypertension, diabetes mellitus, recent smoking history), recently diagnosed during a CT scan with a large left common iliac artery aneurysm measuring 24 cm/17 cm/15 cm, was referred to our clinic for treatment.

**Figure 4:**
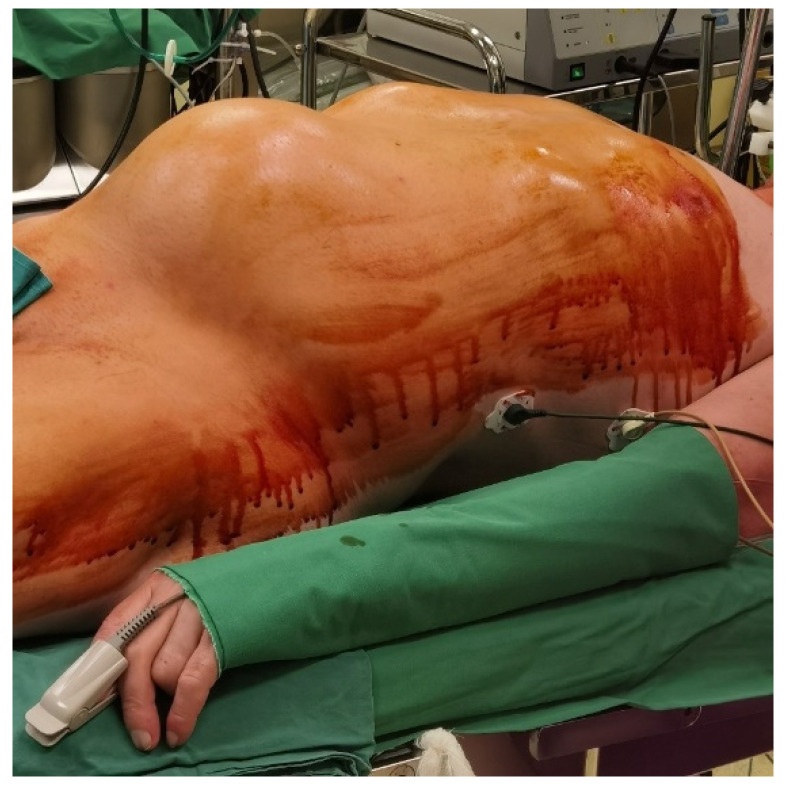
Image of the large abdominal palpable mass.

Physical examination revealed apyrexia, a bilateral vesicular murmur with no added pulmonary sounds, SpO2 = 93% without oxygen, rhythmic cardiac sounds, no cardiac murmurs, HR = 83/min, BP = 135/90 mmHg, and normal bilateral pulses in all limbs. The patient presented weakness and difficulty while moving their left lower limb. The abdomen was distended, without tenderness spontaneously or during palpation, with a giant non-pulsatile mass in the left iliac fossa extending up to the paraumbilical region, as presented in Figure 4. The stool passage was normal but with a slow intestinal transit time (approximately 4 days).

Blood tests revealed no abnormal white cell count, a hemoglobin level of 12.5 g/dL, a Hct level of 36.6%, normal liver and renal parameters, and normal coagulation parameters. Cultures were negative. An ECG revealed a sinus rhythm with 70 bpm, without repolarization abnormalities. The chest X-ray was normal.

The echocardiographic exam showed a slightly dilated left atrium with normal left ventricle, right atrium, and right ventricle. Good systolic function of both ventricles was found and there were no significant valvular modifications.

Coronary angiography showed a dominant right coronary artery and no significant abnormalities.

**Figure 5:**
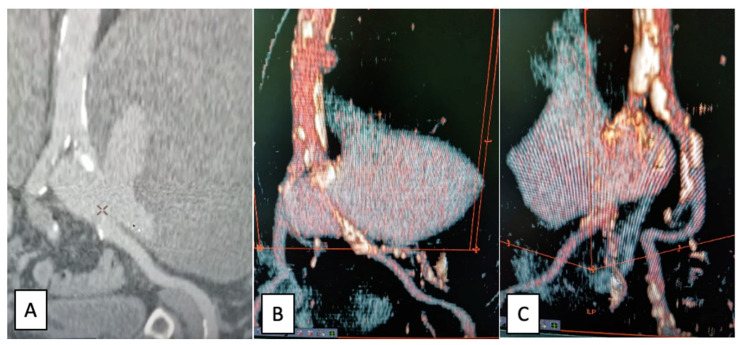
CT images of the left common iliac artery saccular aneurysm: (**A**)—coronal view; (**B**,**C**)—3D reconstruction.

A contrast-enhanced CT scan of the abdomen and pelvis revealed a large left common iliac artery saccular aneurysm (24/17/15 cm) with a large thrombotic burden, no signs of rupture, and no contrast extravasation. Permeability of the distal arterial axes (left external and internal iliac arteries) was present. The digestive tract, superior mesenteric artery, left kidney, uterus, and abdominal aorta were pushed and compressed by the aneurysm. There was also a close contact between the aneurysm and the L3–L5 vertebrae. CT images of the left common iliac artery saccular aneurysm are presented in Figure 5.

**Figure 6:**
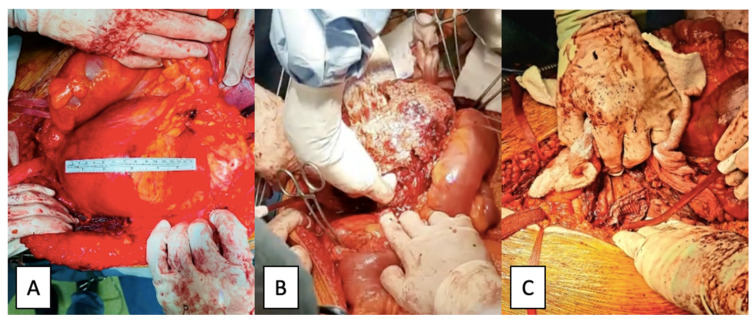
Intraoperative images: (**A**)—pseudoaneurysms content; (**B**)—patch angioplasty (**C**)—left common iliac artery pseudoaneurysm.

As the patient became symptomatic due to the mass effect and the imminent complete rupture of the pseudoaneurysm, in the absence of an available stent graft, the patient underwent emergency open surgical repair using the standard approach of abdominal median transperitoneal lap arotomy. The dissection and isolation of the aorta and both common iliac arteries was laborious because of the modified anatomy secondary to the aneurysmal impingement. Proximal control of the aorta and distal control of both iliac arteries was taken; after clamping the aorta and the iliac arteries, the aneurysm was opened longitudinally and a large retroperitoneal hematoma was evacuated, revealing a parietal tear of the left common iliac artery and, in fact, a false aneurysm that was resolved with a prosthetic Dacron patch sutured to the culprit lesion of the common iliac artery. The intraoperative aspects are presented in Figure 6.

The evolution of the patient was favorable, with extubation at 3 h after admission to the ICU, no neurological impairment, SpO2 = 100%, normal blood gases, hemodynamic stability, normal bilateral pulses in the lower limbs, normal urinary output, and no acidosis. The abdominal drainage was removed in the fourth postoperative day, with resolution of the postoperative ileum.

He made an uneventful recovery and was discharged from the hospital in good condition on the sixth postoperative day.

## 3. Discussion

The incidence of an isolated common iliac artery aneurysm is around 2%. A pseudoaneurysm of the common iliac artery is an even more rare finding [29]. Causes of such an entity may include infection, blunt or penetrating trauma, vasculitis, neurofibromatosis, connective tissue disorders, being iatrogenic after endovascular catheterization, or after surgery and erosion from a malignant tumor [30,31]. They may present as abdominal pain with a palpable mass. Patients may suffer symptoms from the mass effect on surrounding structures with neurologic involvement such as sciatica, lumbosacral pain, proximal leg weakness, and paraesthesia [32]. Cases of patients experiencing foot drop have very rarely been reported [33]. The life-threatening aspect of this pathology is given by the possibility of secondary rupture and bleeding; thus, a prompt diagnosis and treatment are imperative.

An extensive imaging plan is needed for patients with pseudoaneurysms [30,34]. Contrast-enhanced computed tomography is frequently the first imaging technique used for diagnosing a pseudoaneurysm, as it is suitable for detecting large size pseudoaneurysms, but small lesions can easily overlooked [35]. In such cases, angiography is needed, allowing the confirmation of the pseudoaneurysm’s location and an assessment of its eligibility for immediate endovascular treatment if needed [35]. MRI, including MR angiography, could also be used for diagnosing pseudoaneurysms, but the technique is limited by the usually poor clinical condition of the patient, deeming it less useful than angiographic or CT angiographic exams. Color Doppler sonography has been shown to increase the detection of pseudoaneurysms, but its capability of diagnosing them depends on the site of the lesion and the experience of the operator, although the relative low cost and the ability of performing it at the bedside might make it the ideal first-line-examination technique [35].

The endovascular method of treating iliac artery pseudoaneurysms began with using endovascular stents alone [36], arriving now to the implantation of grafted stents [37], or using coils in combination with stents [38]. These approaches eliminated the need, in select cases, for conventional surgical methods, such as patch angioplasty, lateral suturing for narrow-necked pseudoaneurysms, or graft interposition for those with a wider neck, which are procedures accompanied by multiple disadvantages, such as a long hospitalization time, the requirement of general anesthesia, and the possibility for impaired wound healing, and are associated with a higher morbidity and mortality rate [39]. The sturdiness of iliac pseudoaneurysm endovascular repair is still debatable, with a lack of long-term reported outcomes. However, some published studies have compared the open surgical and endovascular repair of isolated iliac artery aneurysms, revealing similar patency in both approaches [40,41]. Still, when pressure-related symptoms are predominant, the surgical approach might be a better option, with a faster reduction in their intensity.

When discussing arterial patches, the trend is towards the biological bovine patch, which is now considered the superior patch. However, the price of the patch makes it unavailable in certain health systems. Moreover, the alternative for biological patches is the porcine patch, which has been deemed inferior to a certain synthetic one, given the fact that it can dilate and calcify in a short amount of time. The synthetic patches are the expanded polytetrafluoroethylene patch (ePTFE) and the knitted and collagen-impregnated polyethylene terephthalate patch (Dacron), of which the generally preferred one is the ePTFE. In certain cases (such as ours), the ePTFE patch can be less useful, as it can be more rigid and, whilst using a 4-0 Prolene suture, the needle holes made can increase the risk of bleeding. [38,39]

Whilst reviewing the reported cases of isolated iliac artery pseudoaneurysms, there were few found. We grouped them by etiology. The most frequent cause was by microbial infection. To name a few, Hsu et al. [42] and Chandler et al. [43] reported two cases of infected left common iliac artery pseudoaneurysms in a 64-year-old man and right external iliac artery pseudoaneurysm respectively secondary to acute appendicitis. The first patient was treated with 14 days of antibiotics, followed by endovascular exclusion of the pseudoaneurysm with the placement of a stent. The second case was treated using a hybrid approach, with embolization of the pseudoaneurysm followed by an extra-anatomic revascularization (a left common femoral to right superficial femoral vein bypass) [42,43]. Pitcher et al. [44] described a case of an unusual mycotic right common iliac artery pseudoaneurysm with methicillin-susceptible *Staphylococcus aureus* (MSSA) of unknown etiology in a healthy 57-year-old man, with no history of infection and no signs of infection at the time of presentation. He underwent pseudoaneurysm exclusion using a covered stent, but approximately two weeks afterwards he developed a fever, the blood cultures were positive for MSSA and he needed a stent explantation, aggressive debridement, and surgical repair [44]. We also found some cases of iatrogenic cause. Taif et al. [31] reported the case of a right common iliac artery pseudoaneurysm 20 years after bladder surgery treated by open aneurysmectomy and iliofemoral bypass. Doleman et al. [45] presented a case of right external iliac artery pseudoaneurysm following balloon angioplasty in a patient with neurofibromatosis type 1 and bilateral pheochromocytoma managed using thrombin injection at the site of the pseudoaneurysm [45]. Two cases of common iliac artery pseudoaneurysm after pancreatic and renal transplant were reported by Leite et al. [46] and Borges et al. [47]. Both patients were managed using endovascular exclusion of the pseudoaneurysm. Only one case of idiopathic left internal iliac artery was found described by Ramakrishnan et al. [32], and it was treated using resection and interposition grafting.

The current article is merely a general review of an underexplored entity and its means of treatment. The current general trend is to use endovascular treatment whenever it is possible as a first line of action, but a surgical approach in selected cases, and possibly a combined approach when the case requires it (stenting endovascular and surgical drainage of the hematoma). The kissing stent method is also a viable option used in experienced centers, but it is a technique that should go through further analysis. All means of treatment should be tailored to the certain needs of the patients and the least invasive treatment should be the first option to reduce the risk of complications and hospitalization, and to bring benefits to the general well-being of the patient [48,49].

## 4. Conclusions

Isolated common iliac artery pseudoaneurysms are an extremely rare finding. They may be asymptomatic or might present as a painful abdominal mass with symptoms from the mass effect on surrounding structures. An extensive imaging plan is needed for patients with pseudoaneurysms. Treatment options include endovascular exclusion with stents alone, implantation of grafted stents, or using coils in combination with stents, or conventional surgical approaches such as patch angioplasty, lateral suturing for narrow-necked pseudoaneurysms, or graft interposition for those with a wider neck. Lately, the preferred method of treatment seems to be the endovascular exclusion, which avoids the multiple disadvantages of the surgical approaches such as a long hospitalization time, the requirement of general anesthesia, and the possibility for impaired wound healing, associated with a higher morbidity and mortality rate. Just a handful of cases have been described in the literature, most of them being mycotic pseudoaneurysms, a few of iatrogenic cause, and only one case being idiopathic.

## Data Availability

Data available on request.
